# Health Services and Economic Impacts of the Limit of Detection in Emergency Department (LEGEND) Rule‐Out Strategy in Australian Emergency Departments: A Stepped‐Wedge Cluster Randomised Trial

**DOI:** 10.1111/1742-6723.70129

**Published:** 2025-09-01

**Authors:** Olivia Dobson, Louise Cullen, William Parsonage, Laura Stephensen, David Brain, Steven Mcphail, Emma Hall, Niranjan Gaikwad, Siegfried Perez, Katrina Starmer, Gregory Starmer, Jaimi Greenslade, the LEGEND investigators, Jaimi Greenslade, Louise Cullen, William Parsonage, Laura Stephensen, Robert Bonnin, David Brain, Emily Brownlee, Virginia Campbell, Brooke Charters, Derek Chew, Gemma Figtree, Vanessa Funk, Gavin Fincher, Niranjan Gaikwad, Emma Hall, Christian Hamilton‐Craig, Ellyse McCormick, Steven M McPhail, Siegfried Perez, Jeremy Rigney, Gregory Starmer, Katrina Starmer, Richard Stone

**Affiliations:** ^1^ Faculty of Health, School of Public Health and Social Work Queensland University of Technology Brisbane Australia; ^2^ Royal Brisbane and Women's Hospital Herston Australia

**Keywords:** acute coronary syndrome, emergency department, healthcare utilisation, high‐sensitivity troponin, resource utilisation

## Abstract

**Objective:**

This study aimed to describe healthcare utilisation and costs associated with the assessment of suspected acute coronary syndrome (ACS) under standard care and to compare these outcomes with the Limit of Detection in Emergency Department (LEGEND) strategy, an accelerated diagnostic pathway identifying low‐risk patients using a single highly sensitive troponin (hs‐cTnI).

**Method:**

A stepped‐wedge cluster randomised trial was conducted in four Queensland hospitals. Each transitioned from standard care (2016 ACS guidelines) to the LEGEND intervention at randomised intervals. Data were collected for index presentations and 6‐month outcomes.

**Results:**

Data were collected from 5347 patients in the standard care phase and 4597 in the LEGEND intervention phase. The intervention reduced mean ED length of stay (−72.0 min, 95% CI: −85.0 to −59.0 min) and inpatient admissions (−2.3%, 95% CI: −4.2% to −0.4%). For low‐risk patients, the intervention further reduced ED length of stay (−97.0 min, 95% CI: −120.5 min to −73.5) and inpatient admissions (−4.2%, 95% CI: −6.9 to −1.6%). Exercise stress testing (EST) utilisation decreased by 3.6% (95% CI: 2.3%–4.9%) overall and 7.7% (95% CI: 5.0%–10.4%) among low‐risk patients during the intervention phase. Total costs decreased from $6849 to $5794 per patient overall, saving $1055 per patient and from $2847 to $2129 per low‐risk patient, saving $718 per patient.

**Conclusions:**

The LEGEND strategy demonstrated reduced resource utilisation and costs compared to guideline‐based ACS assessment, particularly for low‐risk patients. Widespread adoption could improve the efficiency and cost‐effectiveness of ACS assessment in the healthcare system.

## 1 Introduction

Annually, over 500,000 patients are investigated for suspected acute coronary syndrome (ACS) in Australian emergency departments (EDs) [1]. Current ACS assessment uses clinical history, ECGs and serial troponin testing. Most patients are deemed intermediate risk, requiring resource‐intensive assessments and prolonged hospital stays [1, 2]. However, fewer than 15% will ultimately receive a definitive ACS diagnosis [3].

A prior Australian study of ED patients with chest pain reported substantial resource use in suspected ACS assessment [3], underscoring the need for more efficient, cost‐effective care. However, the study's limited 30‐day follow‐up period and restriction to consenting patients presenting during work hours likely underestimated utilisation, reinforcing the need for further research to comprehensively evaluate health service use and costs.

One promising approach to accelerated assessment involves identifying low‐risk patients using a single troponin test. Observational studies have demonstrated that a single highly sensitive troponin (hs‐cTn) value below the limit of detection (LoD) can rule out acute myocardial infarction (AMI) with high negative predictive value (> 99.5%) [4, 5]. These studies recommend further testing in patients presenting within 2 h of symptom onset [5]. The health service and economic impact of early rule‐out strategies for hs‐cTn below the LoD is unknown.

The *L*imit of Detection in *E*mer*gen*cy *D*epartment (LEGEND) strategy combines hs‐cTn concentrations from a single test taken on presentation with shared decision‐making to identify patients who can be discharged without further evaluation in hospital. This study represents the first randomised trial to evaluate health service utilisation and costs for ACS assessment in ED patients using highly sensitive troponin assays as part of the LEGEND strategy. We aimed to describe healthcare utilisation and costs associated with assessment of patients for suspected ACS over 6 months under standard care, and compare these with the LEGEND strategy. It was hypothesised that the LEGEND strategy would reduce healthcare utilisation and costs over a 6‐month period.

## 2 Methods

### 2.1 Study Design

**FIGURE 1:**
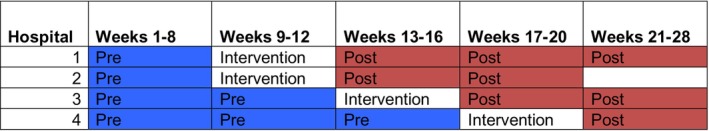
Design of the LEGEND stepped‐wedge cluster randomised trial. *Note:* Blue and red squares represent pre‐intervention and post‐intervention.

This stepped‐wedge cluster randomised trial was conducted in four hospitals in Queensland, Australia. Hospitals were randomly allocated to one of several pre‐specified sequences, each defining the timing at which the hospital would transition from standard care to the LEGEND intervention (Figure 1). The treatment sequences were randomised by a statistician (J.G.) using R software. Each hospital was treated as a cluster and randomly allocated to intervention timing before trial commencement. Blinding of the intervention timing was not possible.

As shown in Figure 1, hospitals began with a usual care phase to capture current practice and outcomes, followed by a 4‐week implementation phase during which education on the clinical practice change was provided to clinical teams. Data were collected for 6 months at all sites, except one where collection ended after 4 months due to COVID‐related disruptions.

The study protocol was approved by a Human Research Ethics Committee (HREC/2018/QRBW/45352) and prospectively registered with the Australia and New Zealand Clinical Trials Registry (ACTRN12618001833257). Reporting followed the Consolidated Standards of Reporting Trials (CONSORT) reporting guidelines extension for stepped wedge cluster randomised trials [6] (Appendix S1).

### 2.2 Inclusion and Exclusion Criteria

Patients were included if they were ≥ 18 years old, and the treating physician deemed that the patient should be investigated for ACS. Exclusion criteria included non‐ACS admissions, transfer from another hospital, pregnancy, prior study enrolment, or unwillingness to be contacted post‐discharge.

### 2.3 Standard Care

In the usual care phase, patients were managed according to their local hospital's documented clinical pathways. All hospitals risk stratified patients using a combination of clinical characteristics, serial troponin testing and serial ECGs. Patients were then referred for further inpatient or outpatient testing. Each site used a pathway that was based on the Australian clinical guidelines for the management of ACS 2016 [1], and the Beckman Coulter Access high‐sensitivity troponin assay.

### 2.4 Intervention

During the intervention, patients with a presentation hs‐cTn result of ≤ 2 ng/L were provided a pictorial representation of their 30‐day risk of ACS. This threshold was chosen as it was the LoD (rounded) for the Beckman Coulter troponin assay. The LoD was chosen rather than a higher cutoff, as this was the only single troponin strategy incorporated in the 2016 National Heart Foundation of Australia and Cardiac Society of Australia and New Zealand Guidelines for the assessment of ACS. A shared decision‐making form was then used to determine whether the patient wished for early discharge or to undergo further investigation. For patients who were discharged, a standardised discharge summary was sent to the patient's primary care physician. An action plan for use in case of recurrent symptoms was incorporated. For patients with a hs‐cTn > 2 ng/L, assessment occurred as per standard care. Patients with ischaemic ECGs were not eligible for early discharge. Patients with pain onset less than 2 h prior to assessment could be included only if they had a hs‐cTn taken 2 or more hours after symptom onset.

Education included face‐to‐face sessions with ED staff about troponin testing, criteria for serial troponin testing and risks and benefits of functional or objective testing for coronary ischaemia. Data were provided on the risks associated with exercise stress testing (EST) and the low specificity of this test for patients at low risk for ACS. Education was reinforced with email communications, and research staff provided ongoing support during the intervention.

### 2.5 Data Collection

Designated research staff collected data throughout the trial. The medical records for every patient who had a troponin test ordered within the ED were reviewed. Research staff identified whether the treating team had investigated patients for suspected ACS. For such patients, information on baseline demographics, cardiac risk factors, prior medical history, ECG results, troponin test results, results from anatomical or functional cardiac testing, ED and hospital length of stay (LOS), disposition information and discharge diagnosis was collected. Six months after the initial presentation, research staff reviewed statewide hospital databases to identify whether the patient had further troponin testing, outpatient cardiac investigations or admissions to a public hospital for suspected ACS assessment. Where appropriate, patients were phoned to identify whether they had visited their general practitioner, a private cardiologist, or a private hospital within 6 months.

### 2.6 Costs

Appendix S2 provides details of how costings were assigned. We used data from the Australian Medical Benefits Scheme [7] of reimbursement to value cardiac testing and other procedures. The value assigned to ED LOS was $226 per hour, a figure based on the previous work by Cullen et al. [3]. Hospital LOS was costed based on the Independent Health and Aged Care Pricing Authority (IHACPA) data [8]. The 2021 average acute cost ($5315 per separation) was used minus costs apportioned to prostheses, operating room, critical care, special procedures and ED. This cost ($3851) was then divided by the average LOS (2.2 days) to provide a daily cost. All costs were then indexed to 2023 costs using the Reserve Bank of Australia inflation calculator.

### 2.7 Data Analysis

Data were analysed using SPSS, version 29.0.1, using an intention‐to‐treat approach. Baseline characteristics were reported using descriptive statistics. To describe the costs associated with suspected ACS assessment, descriptive statistics were reported for investigations performed, cardiac procedures, ED LOS and hospital LOS for any presentations within 6 months. Costs were assigned to these activities as described above, with total costs for each intervention group reported separately for index visits and re‐presentations, as well as a total combining index and re‐presentation costs. Costs per patient were calculated by dividing total costs by the number of patients.

Data were reported based on intervention condition. To assess the impact of the LEGEND assessment strategy on patients categorised as low risk (i.e., baseline hs‐cTnI ≤ 2 ng/L), cost difference analyses were repeated and presented separately for this subgroup. The sample size for this study was large, meaning that statistical testing is likely to identify small differences as statistically significant (even if they are not clinically significant). For this reason, we limited the use of *p* values. Where *p* values were used, we calculated these using chi‐square tests for categorical data and *t* tests for continuous data. Significance was considered at *p* < 0.01.

## 3 Results

### 3.1 Total Patient Cohort Outcomes and Costs

**FIGURE 2:**
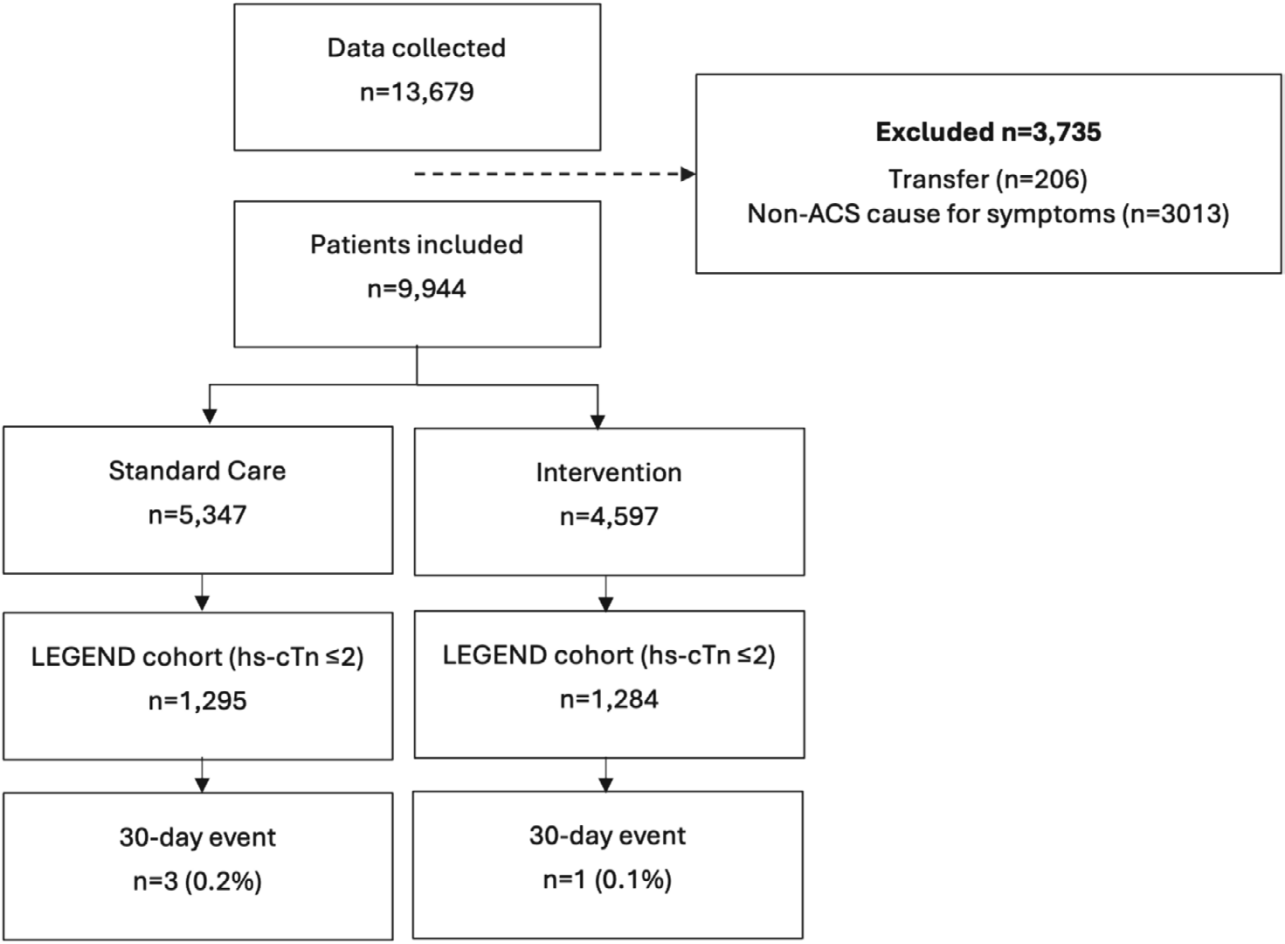
CONSORT diagram for participant flow in the LEGEND trial.

Data were collected from 5347 patients in the standard care phase and 4597 in the LEGEND intervention phase (refer to Figure 2 for CONSORT diagram). The data collection period was 12 August 2019 to 31 July 2020, with follow‐up information obtained for 78.0% of eligible patients. The standard care and intervention cohorts (Table 1) were comparable in sex (50.8% vs. 50.8% male), prior medical history (e.g., prior AMI: 14.2% vs. 12.1%; diabetes: 19.2% vs. 17.5%), median initial troponin concentration (4 [IQR 3–10] vs. 4 ng/L [IQR 2–9]) and median time from arrival to first troponin result (41 [IQR 25–72] vs. 35 min [IQR 22–60]).

**TABLE 1:** Baseline characteristics of standard care and intervention patient cohorts.^a^

Characteristics	Entire cohort		Low‐risk patient cohort	
	Standard care (*n* = 5347)	Intervention (*n* = 4597)	Standard care (*n =* 1295)	Intervention (*n* = 1284)
Age in years, mean (SD)	57.1 (18.2)	56.1 (17.8)	43.6 (14.3)	44.0 (13.9)
Male, *n* (%)	2753 (51.5%)	2335 (50.8%)	470 (36.3%)	451 (35.1%)
Aboriginal and/or Torres Strait Islander, *n* (%)	361 (6.8%)	441 (9.6%)	99 (7.6%)	127 (9.9%)
Presentation ≥ 2 h, *n* (%)	3442 (77.2%)	2910 (78.7%)	879 (78.0%)	855 (77.7%)
Median initial troponin (ng/L) (IQR)	4 (3–10)	4 (2–9)	2 (1–2)	2 (1–2)
Median min to first troponin (IQR)	41 (25–72)	35 (22–60)	44 (28–73)	37 (23–64)
Single troponin testing, *n* (%)	933 (17.5%)	1389 (30.2%)	327 (25.3%)	766 (59.7%)
Risk factors, *n* (%)				
Smoker	1103 (20.6%)	868 (18.9%)	314 (24.3%)	284 (22.1%)
Family history of AMI	814 (15.2%)	649 (14.1%)	290 (22.4%)	245 (19.1%)
Hypertension	2385 (44.6%)	1835 (39.9%)	228 (17.6%)	248 (19.3%)
Dyslipidaemia	2082 (38.9%)	1372 (29.8%)	230 (17.8%)	201 (15.7%)
Diabetes	1028 (19.2%)	803 (17.5%)	108 (8.3%)	115 (9.0%)
Medical history, *n* (%)				
Prior AMI	761 (14.2%)	558 (12.1%)	36 (2.8%)	36 (2.8%)
Prior CAD	1239 (23.2%)	905 (19.7%)	78 (6.0%)	69 (5.4%)
Prior CABG	333 (6.2%)	238 (5.2%)	7 (0.5%)	6 (0.5%)
Prior angioplasty	586 (11.0%)	434 (9.4%)	34 (2.6%)	28 (2.2%)

Abbreviations: AMI, acute myocardial infarction; CABG, coronary artery bypass graft; CAD, coronary artery disease; IQR, interquartile range; SD, standard deviation.

^a^ There were 4747 patients with unknown time of chest pain onset.

Outcome and cost data by intervention group are presented in Table 2. Patients in the intervention phase had a shorter mean ED LOS (mean difference −72.0 min, 95% CI: −85.0 to −59.0 min, *p* < 0.001), a similar hospital LOS (mean difference 0.1 days, 95% CI: −0.29 to 0.51 days, *p* = 0.05) and slightly fewer inpatient admissions (mean probability difference −2.3%, 95% CI: −4.2% to −0.4%, *p* = 0.01), compared to standard care. The average ED and hospital LOS for patients who re‐presented was similar for intervention and standard care arms. However, the proportion of patients who re‐presented and were admitted to an inpatient ward was lower in the intervention phase compared to standard care (mean probability difference −8.7%, 95% CI: −12.2% to −5.2%, *p* < 0.001).

**TABLE 2:** Objective test utilisation, revascularisation, ED and inpatient length of stay, and mean costs for patient index and re‐presentation visits.^a^

	Numbers		Costs			
			Total costs		Costs per patient	
Outcomes	Standard care (*n* = 5347)	Intervention (*n* = 4597)	Standard care	Intervention	Standard care	Intervention
*Index visits*						
Tests						
Ex. stress test	727 (13.6%)	460 (10.0%)	$126,062	$79,764	$24	$17
MPS	242 (4.5%)	169 (3.7%)	$163,568	$114,227	$31	$25
Stress echo	267 (5.0%)	227 (4.9%)	$123,127	$104,681	$23	$23
Echocardiography	1060 (19.8%)	900 (19.6%)	$246,768	$209,520	$420	$46
CTCA	235 (4.4%)	188 (4.1%)	$180,750	$144,600	$34	$31
Angiography	435 (8.1%)	421 (9.2%)	$438,589	$424,473	$82	$92
Cardiac MRI	96 (1.8%)	75 (1.6%)	$47,088	$36,788	$9	$8
PCI	104 (1.9%)	113 (2.5%)	$241,644	$262,556	$45	$57
CABG	29 (0.5%)	29 (0.6%)	$77,837	$77,837	$15	$17
Mean ED LOS (min) (SD)	462 (352)	390 (301)	$9,304,849	$6,752,993	$1740	$1469
Mean inpatient LOS (days) (SD)	4.3 (6.1)	4.2 (6.0)	$16,738,696	$13,172,208	$3130	$2865
Inpatient admissions	1976 (37.0%)	1592 (34.6%)				
Index cost			$27,688,979	$21,379,647	$5178	$4651
*Re‐presentations*						
Patients (% of index visits)	*n* = 1002 (18.7%)	*n* = 739 (16.1%)				
Number of re‐presentations	1824	1135				
Tests						
Ex. stress test	22 (1.2%)	16 (1.4%)	$3815	$2774	$4	$4
MPS	28 (1.5%)	16 (1.4%)	$18,925	$10,814	$19	$15
Stress echo	12 (0.7%)	6 (0.5%)	$5534	$2767	$6	$4
Echocardiography	121 (6.6%)	109 (9.6%)	$28,169	$25,375	$28	$34
CTCA	22 (1.2%)	18 (1.6%)	$16,921	$13,845	$17	$19
Angiography	60 (3.3%)	53 (4.7%)	$60,495	$53,437	$60	$72
Cardiac MRI	8 (0.4%)	5 (0.4%)	$3924	$2453	$4	$3
PCI	19 (1.0%)	20 (1.7%)	$44,147	$48,794	$44	$66
CABG	2 (0.1%)	4 (0.4%)	$5368	$10,736	$5	$15
Mean ED LOS (min) (SD)	267 (215)	273 (184)	$1,826,152	$1,167,121	$1823	$1579
Mean inpatient LOS (days) (SD)	2.7 (5.5)	2.8 (6.4)	$6,930,657	$3,927,392	$6917	$5314
Inpatient admissions	1327 (72.7%)	755 (66.5%)				
Total re‐presentation cost			$8,944,107	$5,265,508	$8926	$7125
Total cost of index + re‐presentation			$36,633,085	$26,645,155	$6851	$5796

Abbreviations: CABG = coronary artery bypass grafting, CTCA = computed tomography coronary angiography, ED = emergency department, Ex. stress test = exercise stress test, LOS = length of stay, MPS = myocardial perfusion scan, MRI = magnetic resonance imaging, PCI = percutaneous coronary intervention, stress echo = stress echocardiography.

^a^ Unless noted otherwise, data are shown as *n* (%).

During the index presentation, EST was performed in 727 patients (13.6%) in the standard care phase and 460 (10.0%) patients in the intervention phase, a reduction of 3.6% (95% CI: 2.3%–4.9%, *p* < 0.001). Index myocardial perfusion scanning (MPS, *p* = 0.03), stress echocardiography (*p* = 0.90), echocardiography (*p* = 0.76), coronary CT angiography (CTCA, *p* = 0.45) and cardiac MRI (*p* = 0.53) were similar between standard care and intervention groups. In the standard care phase, 18.7% (*n* = 1002) of patients re‐presented, compared to 16.1% (*n* = 739) in the intervention group, representing a reduction of 2.6% (95% CI: −4.2% to −1.2%, *p* < 0.001) For re‐presentations, test utilisation and revascularisation procedures were similar in standard care and intervention phases. One exception was for patients who re‐presented to ED with suspected ACS, where echocardiography was performed in 6.6% of re‐presentations in the standard care and 9.6% of re‐presentations in the intervention group (mean probability difference 3.0%, 95% CI: 0.9 to 5.0%, *p* < 0.01).

Total combined costs were $6849 per patient under standard care versus $5794 in the intervention, an average saving of $1055 per patient.

### 3.2 Low‐Risk Patient Cohort Outcomes and Costs

Outcomes and costs for low‐risk patients are presented in Table 3. Approximately one‐quarter of patients had a baseline hs‐cTnI of ≤ 2 ng/L, including 24.2% (*n* = 1295) of patients in the standard care phase and 27.9% (*n* = 1284) in the intervention phase. The groups were comparable in sex (36.3% vs. 35.1% male), prior medical history (e.g., prior AMI: 2.8% vs. 2.8%; diabetes: 8.3% vs. 9.0%) and initial troponin concentration (2 ng/L [IQR 1–2] in both groups). Time from arrival to first troponin result was also similar (44 [IQR 28–73] vs. 37 min [IQR 23–64]).

**TABLE 3:** Objective test utilisation, revascularisation, ED and inpatient LOS and costing data for index and re‐presentation visits of cohort with hsTnI of 2 or less.^a^

Outcomes	Numbers		Costs			
			Total costs		Costs per patient	
	Standard care *n* = 1295	Intervention *n* = 1284	Standard care	Intervention	Standard care	Intervention
*Index visits*						
Ex. stress test	232 (17.9%)	131 (10.2%)	$40,229	$22,715	$31	$18
MPS	20 (1.5%)	15 (1.2%)	$13,518	$10,139	$10	$8
Stress echo	53 (4.1%)	51 (4.0%)	$24,441	$23,519	$19	$18
Echocardiography	115 (8.9%)	98 (7.6%)	$26,772	$22,814	$21	$18
CTCA	52 (4.0%)	38 (3.0%)	$39,996	$29,228	$31	$23
Angiography	25 (1.9%)	23 (1.8%)	$25,206	$23,190	$19	$18
Cardiac MRI	13 (1.0%)	15 (1.2%)	$6377	$7358	$5	$6
PCI	1 (0.1%)	1 (0.1%)	$2324	$2324	$2	$2
CABG	0 (0.0%)	0 (0.0%)	$0	$0	$0	$0
ED LOS (min)						
Mean ED LOS (min) (SD)	427 (343)	330 (259)	$2,082,835	$1,596,012	$1608	$1243
Mean inpatient LOS (days) (SD)	2.2 (3.2)	2.2 (2.3)	$875,468	$632,764	$676	$493
Inpatient admissions	202 (15.6%)	146 (11.4%)				
Index cost			$3,137,165	$2,370,061	$2423	$1846
*Re‐presentations*						
Patients (% of low risk index visits)	*n* = 156 (12.0%)	*n* = 150 (11.7%)				
Number of re‐presentations	300	224				
Ex. stress test	8 (2.7%)	5 (2.2%)	$1387	$867	$9	$6
MPS	2 (0.7%)	0 (0.0%)	$1352	$0	$9	$0
Stress echo	4 (1.3%)	0 (0.0%)	$1845	$0	$12	$0
Echocardiography	8 (2.7%)	8 (3.6%)	$1862	$1862	$12	$12
CTCA	4 (1.3%)	3 (1.3%)	$3077	$2307	$20	$15
Angiography	5 (1.7%)	2 (0.9%)	$5041	$2017	$32	$13
Cardiac MRI	0 (0.0%)	1 (0.4%)	$0	$491	$0	$3
PCI	2 (0.7%)	2 (0.9%)	$4647	$4647	$30	$31
CABG	0 (0.0%)	0 (0.0%)	$0	$0	$0	$0
Mean ED LOS (min) (SD)	193 (169)	221 (150)	$218,090	$186,465	$1398	$1243
Mean inpatient LOS (days) (SD)	0.9 (2.0)	0.9 (1.4)	$312,048	$164,889	$2000	$1099
Inpatient admissions	176 (58.7%)	93 (41.5%)				
Total re‐presentation cost			$549,349	$363,545	$3521	$2424
total cost of index + re‐presentation			$3,686,513	$2,733,606	$2847	$2129

Abbreviations: CABG = coronary artery bypass grafting, CTCA = computed tomography coronary angiography, ED = emergency department, Ex. stress test = exercise stress test, LOS = length of stay, MPS = myocardial perfusion scan, MRI = magnetic resonance imaging, PCI = percutaneous coronary intervention, stress echo = stress echocardiography.

^a^ Unless noted otherwise, data are shown as *n* (%).

Compared to low‐risk patients in the standard care arm, the intervention group had lower mean ED LOS (−97 min, 95% CI: −120.5% to −73.5, *p* < 0.001) and a lower rate of inpatient admissions (−4.2%, 95% CI: −6.9% to −1.6%, *p* < 0.01). Rates of re‐presentation were comparable in the standard care (12.0%) and intervention phase (11.7%, *p* = 0.78).

During their index presentation, 232 (17.9%) low‐risk patients in the standard care phase underwent an EST. In the intervention phase, 131 patients (10.2%) underwent an EST, reflecting a 7.7% (95% CI: 5.0%–10.4%, *p* < 0.001) reduction in EST utilisation. There were no differences between standard care and intervention arms for any other cardiac test, and testing performed during re‐presentations did not differ by intervention arm.

Total combined costs were $2847 per low‐risk patient under standard care, compared to $2129 in the intervention condition, representing an average saving of $718 per patient.

## 4 Discussion

This study is the first to comprehensively evaluate healthcare utilisation and costs for Australian ED patients presenting with symptoms of suspected ACS, comparing standard care with the LEGEND accelerated diagnostic pathway.

Current ACS assessment involves high testing rates, prolonged stays and frequent re‐presentations, averaging $6850 per patient. Despite minimal AMI risk [4, 5], resource use remained high for low‐risk patients, with almost one in five undergoing EST and demonstrating extended ED stays and increased hospital admissions and re‐presentations, costing $2847 per patient on average. These findings extend previous research by including all ED presentation cases and 6‐month follow‐up data, revealing that the costs of investigating suspected ACS based on the 2016 guidelines are higher than previously estimated for Australian ED cohorts [3].

Results from this study demonstrate that the 2016 guideline‐based process for assessing ACS is resource‐intensive and inefficient, despite fewer than 15% of patients ultimately receiving a definitive ACS diagnosis [3]. This inefficiency contributes to overcrowding and exposes patients to unnecessary risks [9, 10]. The LEGEND strategy provides an alternative approach to streamline this process by identifying low‐risk patients to be discharged without further testing using shared‐decision making based on a single hs‐cTnI result. The intervention reduced EST use, ED time and admissions, with larger decreases for low‐risk patients, contributing to cost savings of $1,055 per patient for the total cohort and $718 per low‐risk patient.

## 5 Limitations

Some limitations must be acknowledged in interpreting these results. At one hospital, data collection was restricted to 4 months due to the COVID pandemic, which affected both ED presentations and data collection. Additionally, there are likely to be costs that are not included in the costing exercise. For example, surgical costs for transferred ACS patients were not included, potentially underestimating total costs. However, as this trial was randomised, these costs are likely to be similar in the two groups. It should be noted that while reduced testing and LOS were the primary reasons for a reduction in cost in the LEGEND arm, there is no agreed value in the literature for costing ED and hospital LOS. Utilising a different cost for LOS would result in a different estimate between treatment arms.

This study implemented the LEGEND strategy as part of standard care and study data were collected from patients' medical records. As such, the specific factors contributing to reduced LOS (e.g., troponin testing versus reduced EST) could not be fully determined. However, with most of the hospitals undertaking outpatient ESTs, the reduction in LOS is likely due to single troponin testing. Patient response to shared decision‐making or physician non‐compliance with the protocol could also not be assessed.

## 6 Conclusion

In conclusion, the 2016 guideline‐based process for assessing suspected ACS is resource‐intensive and costly. The LEGEND strategy offers a viable alternative, reducing resource utilisation and costs, particularly for low‐risk patients. Widespread implementation could significantly improve the cost‐efficiency of suspected ACS assessment pathways to benefit both patients and the Australian healthcare system.

## Conflicts of Interest

The authors declare no conflicts of interest.

## Supporting information


**Appendix S1:** Supporting Information.

**Appendix S2:** Reference costs used in the study.

## Data Availability

The data that support the findings of this study are available on request from the corresponding author. The data are not publicly available due to privacy or ethical restrictions.
