# Supplementary material for: Health Services and Economic Impacts of the Limit of Detection in Emergency Department (LEGEND) Rule‐Out Strategy in Australian Emergency Departments: A Stepped‐Wedge Cluster Randomised Trial
Source: Emerg Med Australas. 2025 Sep 1;37(5):e70129. doi: 10.1111/1742-6723.70129 (PMC12402291; doi:10.1111/1742-6723.70129)
Supplement: Supplementary file 2 — Appendix S2: Reference costs used in the study. [file EMM-37-0-s002.docx]

**Supplementary Material**

**Appendix 2**: Reference costs used in the study

|  |  |  |
| --- | --- | --- |
| Item | **Cost** | **Source/Reference** |
| *Tests* |  |  |
| Exercise Stress Test | $173.40 | MBS Item 11729 |
| MPS | $675.90 | MBS Item 61324 |
| Stress Echocardiography | $461.15 | MBS Item 55141 |
| Echocardiography | $232.80 | MBS Item 55133 |
| CTCA | $769.15 | MBS Item 57364 |
| Angiography | $1007.25 | MBS Item 38244 |
| Cardiac MRI | $490.50 | MBS Item 63385 |
| *Revascularisation* |  |  |
| PCI | $2,323.50 | MBS Item 38313 |
| CABG | $2,684.05 | MBS Item 38502 |
| *ED LOS* |  |  |
| Per hour | $226 | Cullen et al. (3)  Inflated to 2023 costs using RBA calculator |
| *Inpatient LOS* |  |  |
| Per day | $1,970 | Independent Health and Aged Care Pricing Authority (IHACPA) (7). |
